# Chemiluminescence Detection of Hydrogen Peroxide with a Polymer of an Intrinsic Microporosity Solid State Emitter

**DOI:** 10.1021/acsapm.6c00847

**Published:** 2026-05-04

**Authors:** Supharada Phokhabut, Tinakorn Kanyanee, Michael Zachariadis, Silvia Martinez Micol, Philip J. Fletcher, Mariolino Carta, Dominic Taylor, Neil B. McKeown, Marco Caffio, Oliver Matys, Frank Marken

**Affiliations:** † Department of Chemistry, 1555 University of Bath, Claverton Down BA2 7AY, U.K.; ‡ Department of Chemistry, Faculty of Science, 26682 Chiang Mai University, Chiang Mai 50200, Thailand; § Materials Science Research Center, and Center of Excellence for Innovation in Chemistry, Faculty of Science, Chiang Mai University, Chiang Mai 50200, Thailand; ∥ Imaging Facility, University of Bath, Bath BA2 7AY, U.K.; ⊥ Faculty of Science and Engineering, Department of Chemistry, Swansea University, College of Science, Grove Building, Singleton Park, Swansea SA2 8PP, U.K.; # Instituto de Síntesis Química y Catálisis Homogénea, CSIC-Universidad de Zaragoza, C/Pedro Cerbuna 12, Facultad de Ciencias, Zaragoza 50009, Spain; ¶ EaStCHEM, School of Chemistry, 3124 University of Edinburgh, Joseph Black Building, David Brewster Road, Edinburgh, Scotland EH9 3JF, U.K.; ∇ 645032 Integrated Graphene Ltd., Euro House, Wellgreen Place, Stirling FK8 2DJ, U.K.

**Keywords:** electrochemiluminescence, chemiluminescence, energy transfer, microporous host, reagentless sensing, hydrogen peroxide

## Abstract

The intrinsically microporous polymer PIM-1 provides a highly porous and simultaneously fluorescent and emissive host structure for analytical processes. Trichlorophenoloxalate (TCPO; a reagent for excited state intermediate formation with H_2_O_2_) has been embedded into PIM-1 (the microporous host) by codeposition. TCPO reacts with the imidazole buffer, and traces of hydrogen peroxide diffuse into the microporous host to give an excited-state intermediate and energy transfer to the fluorescent PIM-1. This causes effective (electro)­chemiluminescence (ECL or CL) emission in the solid state for microporous films deposited on graphene foam (ECL) or for films on filter paper (CL) with diffusion-limited (Cottrellian) signal decay. On graphene foam electrodes/substrates, the formation of hydrogen peroxide from electrochemical oxygen reduction triggers electrochemiluminescence (ECL). On filter paper substrates with PIM-1/TCPO films, direct exposure to hydrogen peroxide triggers chemiluminescence (CL) emission spectra (equivalent to PIM-1 fluorescence spectra). Hydrogen peroxide-mediated detection of glucose is demonstrated and suggested as an effective/potentially reagentless analytical method for a broader range of applications linked to quantitative H_2_O_2_ analysis.

## Graphical abstract

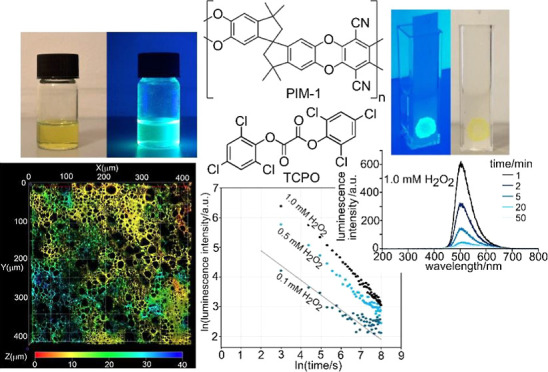

## 1 Introduction

Polymers of intrinsic microporosity^1^ (PIMs) have been developed initially for gas separation,^2,3^ gas sensing,^4^ and gas adsorption^5^ into micropores (with typically 1 nm diameter), which are generated in the solid state by poor packing of molecularly rigid polymer strands.^6^ These polymer materials have now found a much broader range of applications, including processes in liquid media.^7–10^ The emerging potential of PIMs in electrochemical^11^ and in analytical sensing applications has been reviewed.^12^

One of the prototypical PIMs is PIM-1 (see molecular structure in Figure 1A) with strong green-yellow fluorescence^13^ and opportunities for optical sensor applications.^14–16^ PIM-1 has been investigated by N_2_ gas adsorption (BET) analysis and shown to have a density of typically 0.9–1.4 g cm^–3^ and an average pore size of 760– 875 m^2^ g^–1^.^17^ PIM-1 was processed further into hierarchically porous materials^18^ and into chemically modified microporous structures.^19,20^ Here, PIM-1 is demonstrated to act as an emissive host for chemiluminescence. New types of chemiluminescence applications based on PIM-1 fluorescence are suggested with bis­(2,4,6-trichloro-phenyl)­oxalate (TCPO) immobilized into the microporous polymer host to produce excited state intermediates within the microporous material to then trigger fluorescent light emission. The interplay of diffusion of analyte into micropores and time-dependent luminescence is investigated.

**Figure 1:**
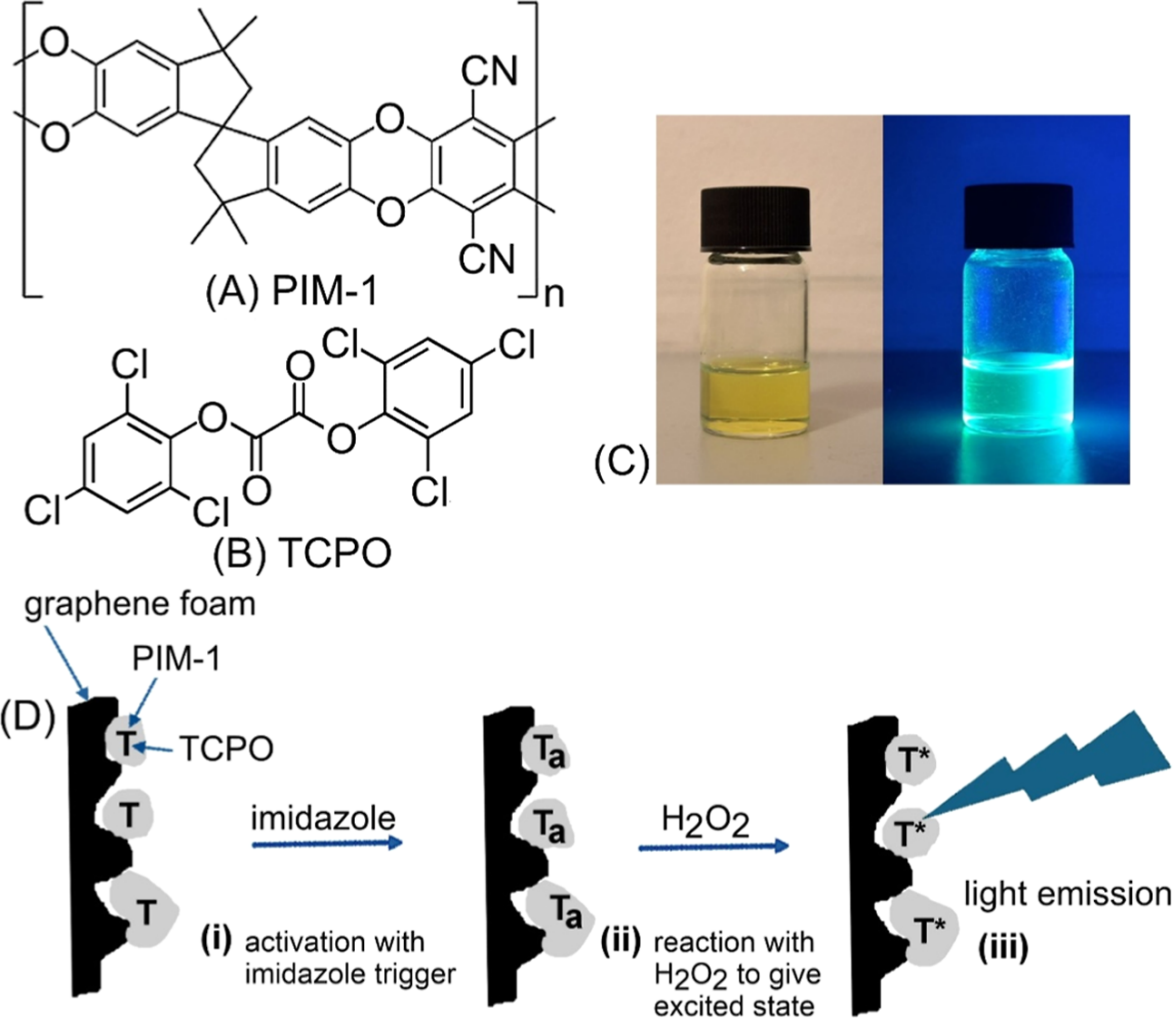
Molecular structures of (A) PIM-1 or (C_29_H_20_N_2_O_4_)_*n*_ with MW 460.4 g mol^–1^ monomer and (B) bis­(2,4,6-trichlorophenyl)­oxalate (or TCPO, C_14_H_4_Cl_6_O_4_, and MW 448.9 g mol^–1^). (C) Photographs showing PIM-1 natural color in chloroform (10 mg cm^–3^) and fluorescence in UV light. (D) Schematic of the proposed reaction pathway for TCPO (T) in PIM-1 interacting with imidazole to give the activated state (*T*_a_) (i), then reacting with H_2_O_2_ to give an excited state intermediate (T*) (ii), and then transferring energy to PIM-1 causing luminescence (iii).

Although fluorescent PIMs have been reported for chiral analysis^21^ and for electrical detection of iodine,^22^ there is very little previous work on emissive sensing in these intrinsically microporous polymer materials. In order to provide an effective emissive sensor interface, there is considerable interest in combining microporosity and chemiluminescence.^23,24^ In the past, light-emitting molecules have been embedded into polymers of intrinsic microporosity^14^ to allow interaction/sensing with vapor-phase analytes. In a recent study, the emission from a combination of PIM-1 and fluorescent carbon nanodots was exploited for electro-chemiluminescence^25,26^ (ECL) detection of citrate as a biomarker for cancer.^27^ For PIM-1 nanoparticles deposited onto tin-doped indium oxide (ITO) electrodes, direct (reagent-free) electro-luminescence was reported possibly linked to excited state energy transfer from products formed at the anode at positive applied potentials.^28^

The reagent bis­(2,4,6-trichlorophenyl)­oxalate (TCPO and C_14_H_4_Cl_6_O_4_, see molecular structure in Figure 1B) has been commonly employed in ECL/CL assays for the detection of hydrogen peroxide.^29^ In emulsion systems, the TCPO can be solubilized (together with perylene dye as the emitter) to react with hydrogen peroxide in aqueous media.^30^ It has been proposed that the trichlorophenylate leaving group is replaced *in situ* by imidazole (the “trigger reagent”^31^) to then allow enhanced luminescence due to attack by H_2_O_2_. Although TCPO can form excited state reaction products with hydrogen peroxide, it is not able to emit photons. Dye emitters such as rhodamine B,^32^ rubrene,^33^ or quantum dots^34^ are added to provide the emission pathway. Here, TCPO is embedded into the fluorescent microporous PIM-1 host, and energy transfer is enabled with emission from PIM-1 (Figure 1C). There is no need for added dyes, and the process occurs within the solid state, with H_2_O_2_ having to diffuse into the microporous environment. Figure 1D summarizes the proposed reaction pathway for graphene foam substrates. Graphene foam electrodes are useful in analytical electrochemistry^35^ and have recently been employed in combination with PIM-1 microporous deposits.^36^ In combination with PIM-1 deposits, the graphene foam exhibited enhanced reactivity toward oxygen and accelerated formation of hydrogen peroxide, H_2_O_2_.^37^

In this report, both the fluorescence and (electro)­chemiluminescence caused by H_2_O_2_ are compared and shown to be spectrally identical (PIM-1 is the emitter in all cases). Fluorescence microscopy is employed to map out the distribution of fluorescent PIM-1 within a porous graphene foam structure or on a filter paper substrate. Time-dependent data suggest diffusion limitations within the microporous host and mixed diffusion-reaction kinetic control. The heterogenization and reactivity of TCPO in the fluorescent host PIM-1 is investigated/demonstrated and optimized. Hydrogen peroxide generated at graphene foam electrodes is shown to cause (electro-)­chemiluminescence (ECL) in the presence of TCPO and PIM-1 (see the simplified mechanism in eqs 1 and 2).
1

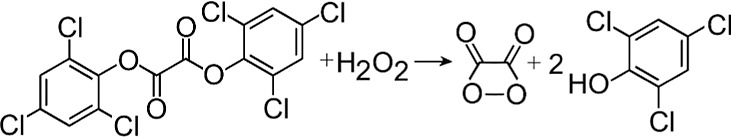



2

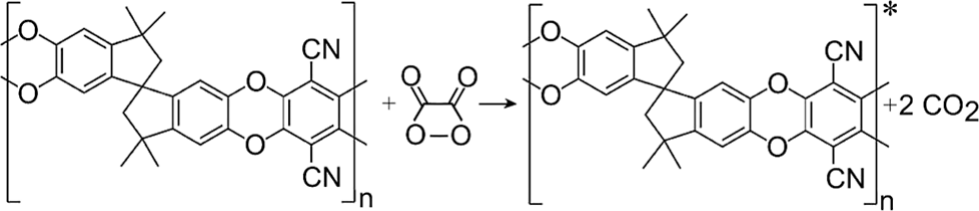

In glucose oxidase-based enzymatic assays, hydrogen peroxide formation is shown to trigger glucose concentration-dependent solid-phase chemiluminescence (CL) emission for PIM-1/TCPO films on strips of filter paper. The effects of diffusion time and H_2_O_2_ concentration on the CL emission intensity are quantified and rationalized with the help of a computational diffusion-reaction model. Future applications are proposed for reagentless sensing or biosensing.

## 2 Experimental Section

### 2.1 Chemicals

Polymer PIM-1 (2,3,5,6-tetrafluorophthalonitrile-3,3,3′,3′-tetramethyl-1,1′-spirobisindane-5,5′,6,6′-tetrol copolymer, Sigma-Aldrich 918768, monomer molecular weight 460 g mol^–1^, molecular weight typically 70 KD) was prepared following a literature procedure.^38^ Chloroform, imidazole, bis­(2,4,6-trichlorophenyl)­oxalate or TCPO (Aldrich 75707), glucose oxidase (G2133, Aldrich), d-glucose, 30% H_2_O_2_, Na_2_HPO_4_, NaH_2_PO_4_, and NaOH were obtained in analytical grade from Aldrich or Fisher Scientific and used without further purification. Solutions were prepared with filtered/deionized water of resistivity of 18.2 MΩ cm (at 20 °C) from a Thermo Fisher filter system.

### 2.2 Instrumentation

A computer-controlled Ivium Compactstat instrument (Ivium, The Netherlands) was employed for electrochemical measurements coupled to electrochemiluminescence (ECL) detection. A photomultiplier tube (PMT, PMM02, Thorlabs UK) was held at an 800 V accelerator voltage by using a programmable function generator (TTi, TG1304, Thurlby Thandar Instruments Ltd.) or the Ivium interface box. The photocurrent produced at the PMT was converted to a voltage signal and fed into the external input channel of the computer-controlled potentiostat.

A three-electrode system was used in a homemade electrochemical cell with a 4 cm gap from the electrode surface to the PMT detector. Graphene foam electrodes were obtained from Integrated Graphene Ltd.^39^ Electrochemical measurements were performed with a single droplet (approximately 50 μL) placed onto the graphene foam electrode. The working electrode was graphene foam (4 mm diameter disk, approximately 40 μm thick) with a counter electrode (a 1 mm wide graphene foam ring) and a printed Ag/AgCl reference electrode. Scanning electron micrographs were obtained with a SU3900 large chamber variable pressure scanning electron microscope with an attached Oxford Instruments Ultim Max 170 mm^2^ low kV energy-dispersive X-ray analyzer. Optical fluorescence microscopy was performed with a Zeiss LSM 880 confocal system with Airyscan and Multiphoton laser. Attenuated total reflection–Fourier transform infrared (ATR–FTIR) spectra were obtained with a Nicolet Summit X instrument (Thermo Scientific, UK). Luminescence spectroscopy data were obtained with a Cary Eclipse fluorescence spectrometer (parameter settings: Bio/Chemi-luminescence; scan setup: emission range 200–800 nm, excitation slit 20 nm; scan control: scan rate 600 nm/min, averaging time 0.05 s, data interval 0.5 nm, gate time: 50 ms; PMT detector voltage: high (800 V); cycle mode: 50 cycles, time 1 min) employing a standard 1 cm path length quartz cuvette.

### 2.3 Procedures

#### 2.3.1 Deposition of TCPO and PIM-1 on Graphene Foam

Both PIM-1 and TCPO were dissolved, each in chloroform (10 mg cm^–3^). When premixing these two solutions, a precipitate formed. Therefore, premixing was avoided, and PIM-1 was first drop-cast onto the graphene foam electrode, and the deposited layer was allowed to dry under ambient conditions. Subsequently, TCPO solution was drop-cast onto the precoated PIM-1 layer (combining with the redissolved PIM-1), followed by drying to yield a composite film. Typically, 30 μg each was deposited to give a 60 μg composite with a 1:1 weight ratio. Figure S1 shows FTIR data for the graphene foam and for the coated foam, indicating diagnostic peaks in the mixed deposit as “P” form PIM-1 and “T” for TCPO. Figure SI2 shows scanning electron microscopy (SEM) images for graphene foam without and with coating of PIM-1/TCPO. Changes in morphology due to the coating are very subtle and difficult to see, but energy-dispersive X-ray spectroscopy (Figure SI3) does reveal the additional presence of chlorine on the surface (compare the background in Figure SI4). In cross-sectional SEM/EDS mapping, the presence of chlorine (from PIM-1/TCPO) can be seen in patches at the surface (Figure SI5).

Fluorescence imaging has been employed (Figure 2) to show that both (A) bare graphene foam and (B) PIM-1/TCPO-coated graphene foam give characteristic emission signatures. Bare graphene foam exhibits a relatively weak emission, which occurs at about 425 nm (with 405 nm laser excitation; Figure 2B). When coating the graphene foam with 60 μg of PIM-1/TCPO (1:1), the emission is enhanced by orders of magnitude with a peak at 504 nm mainly due to PIM-1 (*vide infra*). The pore structure is clearly revealed with depth indicated by color. The film thickness is approximately 40 μm for the working electrode (see Figure 2C; color indicating depth), and therefore, all of the graphene foam is coated in PIM-1/TCPO and actively emitting fluorescence (Figure 2D). Figure 2E shows a photograph of the electrode.

**Figure 2:**
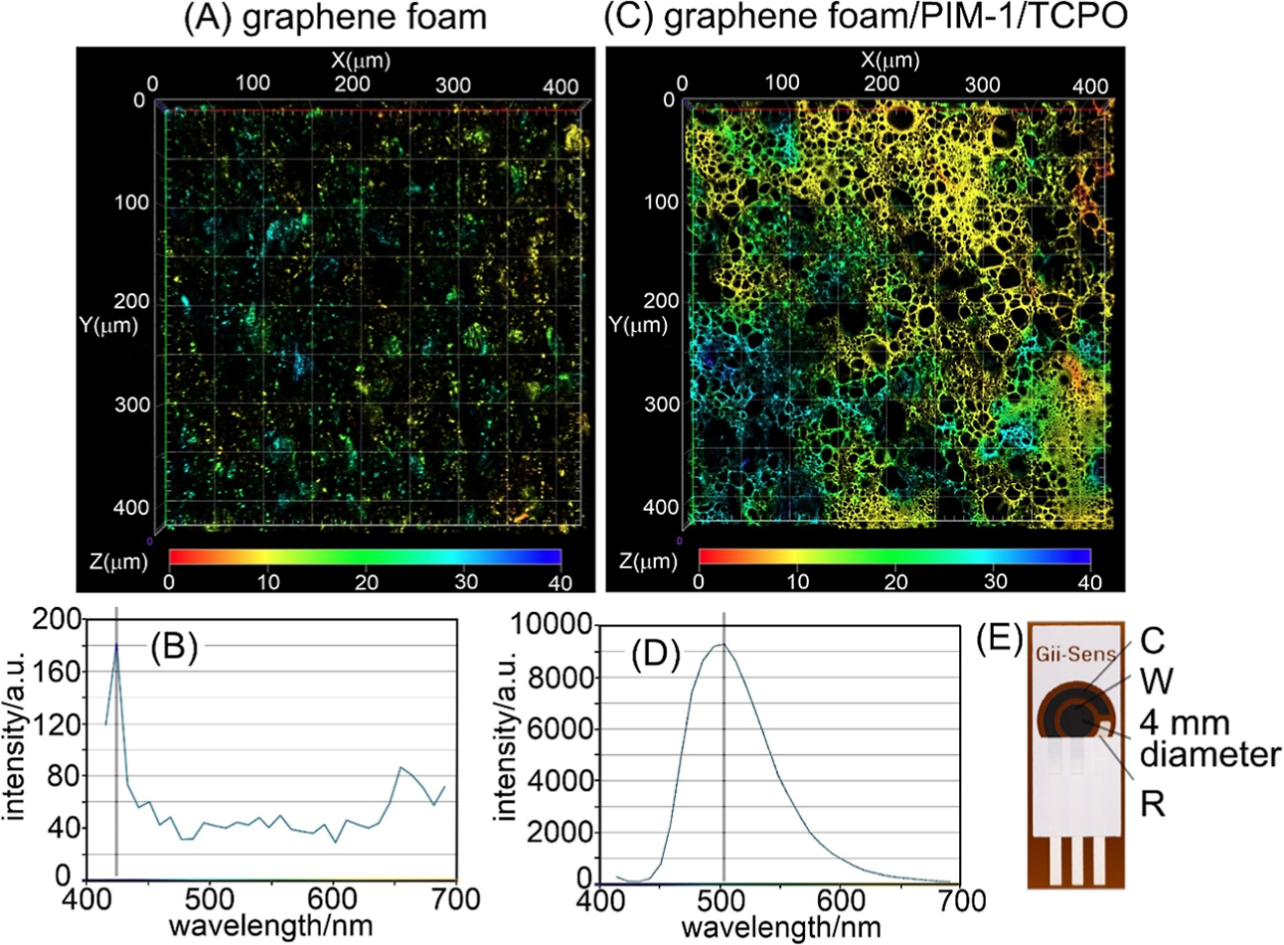
Fluorescence microscopy images (in 3D; showing depth as color), and fluorescence spectra for (A,B) bare graphene foam and (C,D) PIM-1/TCPO-coated graphene foam. (E) Photograph of the graphene foam electrode with counter electrode C, working electrode W, and pseudo-Ag/AgCl reference electrode R.

#### 2.3.2 Deposition of TCPO and PIM-1 on Filter Paper

In order to study chemiluminescence of PIM-1/TCPO in the presence of hydrogen peroxide without any electrochemical processes, a 60 μg deposit of 1:1 PIM-1/TCPO was formed on a strip of filter paper (Whatman 1; spot size typically 7 mm diameter; see Figure 3; see Figure SI1 for FTIR data). The fluorescence microscopy image in Figure 3E shows the three-dimensional distribution of the emissive deposit, in particular, in bigger pores in the filter paper.

**Figure 3:**
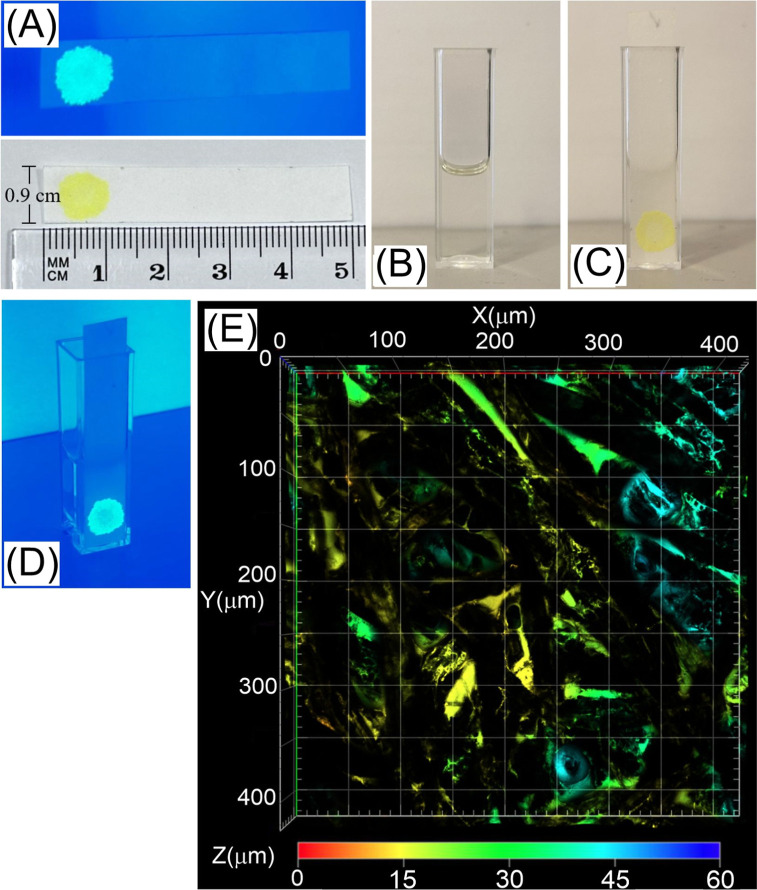
Photographs of (A) filter paper with 60 μg PIM-1/TCPO (1:1) coating in daylight and under UV, (B) cuvette for luminescence measurements, (C) cuvette with filter paper inserted to give emission toward the outside, and (D) emission under UV. (E) Fluorescence microscopy images (in 3D; showing depth as color) for PIM-1/TCPO on filter paper.

In SEM images (Figure SI6), the presence of the PIM-1/TCPO coating on filter paper is difficult to observe, in part due to the microporous and amorphous polymer giving poor contrast. EDS data (Figures SI7 and SI8) clearly show that chlorine atoms associated with TCPO are detected, although nitrogen atoms are present before/after coating in similar amounts. A cross-sectional SEM and EDS data set is shown in Figure SI9. The presence of chlorine at the surface of the filter paper is revealed in Figure SI9E. The thickness of localized coatings could be in the range of a few micrometers, but this extends into the filter paper structure.

## 3 Results and Discussion

### 3.1 Observation and Optimization of Electrochemiluminescence (ECL) from Porous PIM-1/TCPO Deposits

Initially, a deposit of PIM-1 and TCPO (typically 60 μg in total on the geometric electrode area 1.2 × 10^–5^ m^2^) on graphene foam substrates is investigated when immersed into 0.1 M imidazole buffer at pH 7. Figure 4A shows cyclic voltammetry data for three different combinations of materials: (i) PIM-1/TCPO 1:5, (ii) PIM-1-TCPO 5:1, and (iii) PIM-1/TCPO 1:1. In all three cases, small anodic features (unidentified) are observed at 0.6 V vs pseudo-Ag/AgCl and 1.0 V vs pseudo-Ag/AgCl with a higher anodic current at more positive potentials. During the oxidation of the deposit on the graphene foam electrode, there is no ECL emission (Figure 4B). However, when scanning the electrode potential into the oxygen reduction, it was −0.7 V vs pseudo-Ag/AgCl, and a clear light emission event can be observed. The highest level of light emission occurs with PIM-1/TCPO in a 1:1 weight ratio (see voltammograms in Figure 4B). The inset summarizes ECL data for a wider range of compositions.

**Figure 4:**
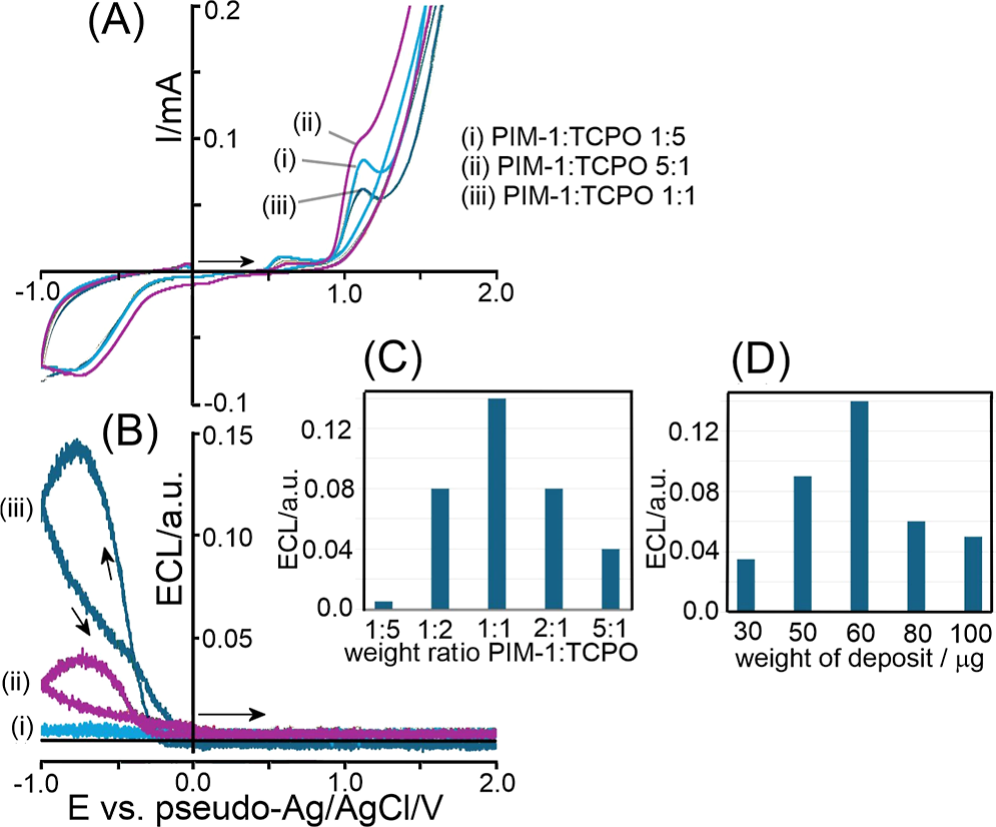
(A) Cyclic voltammograms (current versus potential) and (B) cyclic voltammograms (PMT voltage 800 V; ECL signal versus potential; scan rate 0.02 V s^–1^; in 1 M imidazole buffer pH 7) for a graphene foam electrode coated with 60 μg of (i) PIM-1/TCPO 1:5, (ii) PIM-1/TCPO 5:1, and (iii) PIM-1/TCPO 1:1. Inset (C) Bar graph of ECL intensity versus weight ratio. Inset (D) Bar graph of ECL intensity versus total weight of deposit (1:1).

Figure 4D summarizes the effect of the total amount of PIM-1/TCPO (at a 1:1 ratio) on the graphene foam. An optimum is reached at approximately 60 μg indicative of higher amounts of deposit essentially blocking parts of the electrode or pores. When the solution pH is varied, the oxygen reduction peaks shift more negative with higher pH (Nernstian), but the ECL intensity does not change dramatically in the range from pH 7 to pH 9 (see Figure SI10). The strongest ECL emission occurs at pH 7, which coincides with the p*K*_a_ for the imidazole buffer (p*K*_a_ = 6.9 at 25 °C^40,41^ ). The effect of the imidazole buffer concentration has not been evaluated in more detail, but the involvement of imidazole as a “trigger reagent” suggests that both the buffer concentration and pH will affect the reaction (*vide infra*).

Figure 5A shows cyclic voltammograms and voltammograms with the detection of hydrogen peroxide in every subsequent potential cycle. However, the electro-luminescence intensity decays. One possible reason for this decay (tentatively assigned) could be the gradual formation of the “exhausted zone” where TCPO has been consumed (Figure 5B). This causes the intensity of the electro-chemiluminescence to gradually decrease (*vide infra*) as reagents need to diffuse deeper into the microporous host. A pictorial description of the process is shown in Figure 5B. The internal concentration *c*_TCPO_ = 1 M has been estimated based on the PIM-1/TCPO with a 1:1 composition with an assumed approximate solid-state density of 1 g cm^–3^. The diffusion coefficient for H_2_O_2_ is likely to be similar or lower compared to that of O_2_ in PIM-1, which is in the order of *D*_H_2_O_2__ = 10^–13^ m^2^ s^–1^.^42^ Under these conditions, the diffusion layer would progress into the polymer film with $\delta_{diffusion}=\sqrt{\pi Dt}$ , resulting in a diffusion layer of approximately 2 μm in 20 s (consistent with a thin film deposit). The reaction with TCPO will happen in a reaction layer that is within the diffusion layer (*vide infra*).

**Figure 5:**
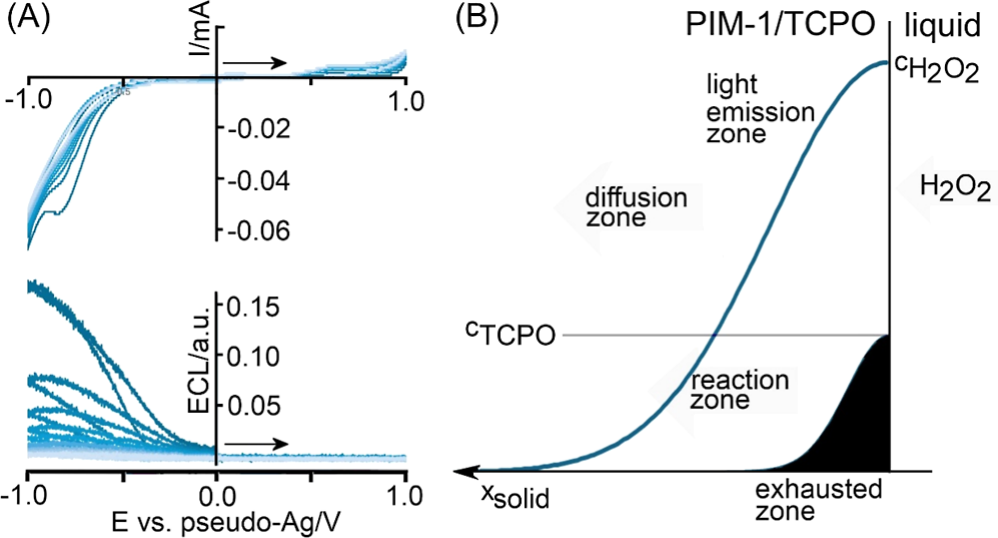
(A) Continuous cycling cyclic voltammograms and simultaneous voltammograms (scan rate 20 mV s^–1^) indicative of gradual loss of ECL emission. (B) Illustration of the formation of a diffusion zone and a reaction zone (not to scale) linked to photoluminescence in the solid (depletion of TCPO indicated as black exhausted zone).

### 3.2 Observation and Optimization of Chemiluminescence (CL) Spectra from Porous PIM-1/TCPO Deposits on Filter Paper

In order to broaden the range of potential applications (including direct hydrogen peroxide detection), the PIM-1/TCPO deposit was applied to a filter paper substrate and then placed in a standard cuvette for luminescence spectroscopy (Figure 3). Typically, PIM-1 and TCPO were applied from a chloroform solution (30 μg each, sequentially to give a mixed deposit), and the resulting distribution of fluorescent material is shown in Figure 3. Figure SI6 shows topographical SEM data, identifying mainly the fibrous substrate. Evidence for the coating is seen in SEM/EDS cross-sectional data (Figure SI9), where the chlorine distribution suggests localized film deposits of a few micron thickness. The best evidence for the presence of the PIM-1/TCPO film is obtained from fluorescence data. Figure 6 shows a comparison of fluorescence spectra of (i) PIM-1/TCPO on dry filter paper, (ii) PIM-1/TCPO on graphene foam in 0.1 M imidazolium buffer (pH 7), and (iii) PIM-1/TCPO on dry graphene foam. A fluorescence peak at approximately 504 nm (for both dry and wet conditions) is consistent with previously reported PIM-1 fluorescence.^43^

**Figure 6:**
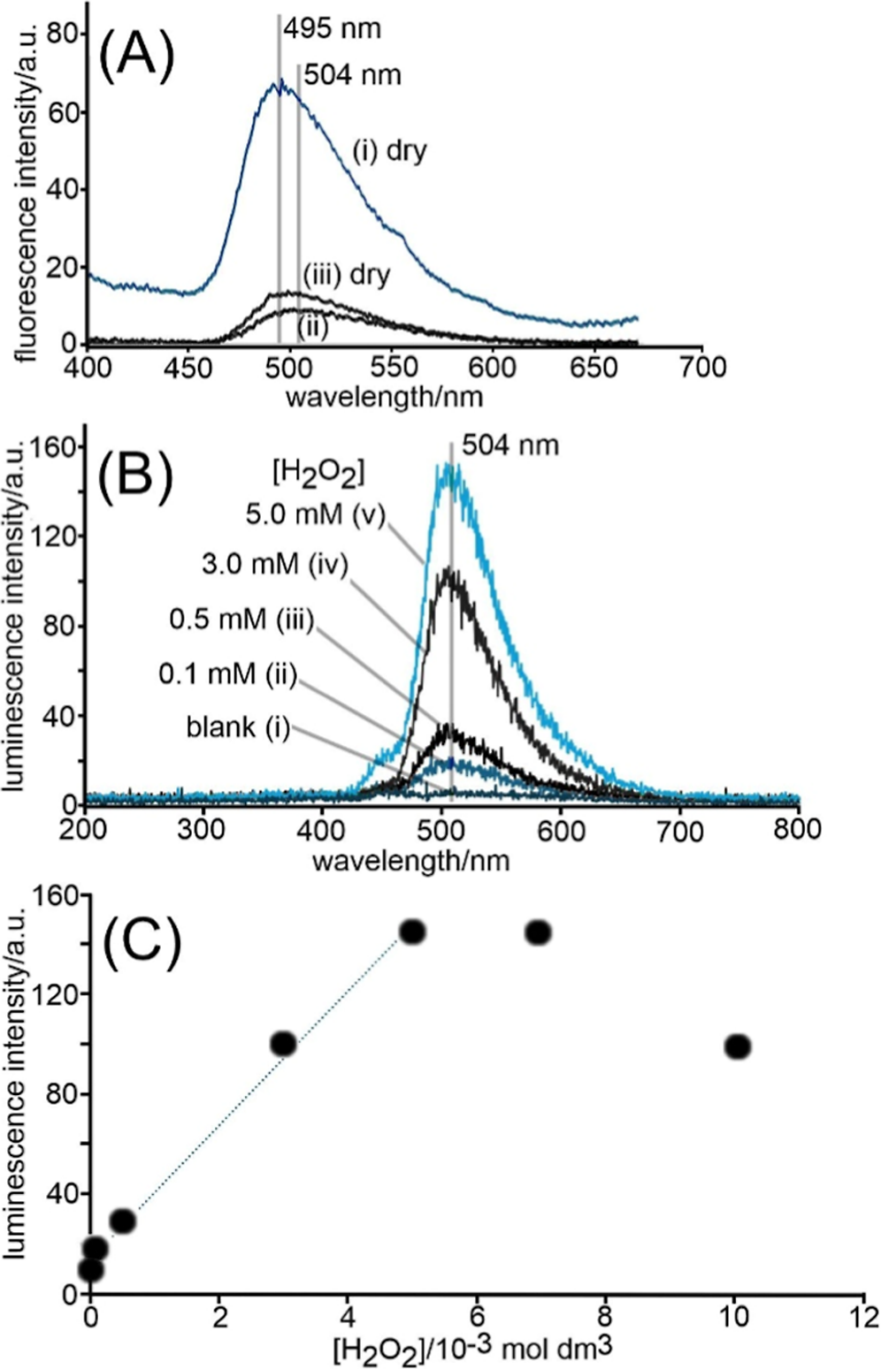
(A) Comparison of fluorescence spectroscopy data for (i) PIM-1/TCPO on dry filter paper, (ii) PIM-1/TCPO on filter paper immersed in 0.1 M imidazole buffer pH 7, and (iii) PIM-1/TCPO on dry graphene foam. (B) Sequential measurement of chemiluminescence (using a single filter paper) as a function of hydrogen peroxide concentration in a 0.1 M imidazole buffer pH 7. (C) Plot of chemiluminescence intensity versus hydrogen peroxide concentration in 0.1 M imidazole buffer pH 7 using a single filter paper (initial increase due to increased hydrogen peroxide concentration and decrease due to exhaustion of TCPO in the emission process).

The chemiluminescence emission spectrum was recorded for PIM-1/TCPO on filter paper immersed in 0.1 M imidazolium buffer at pH 7 and as a function of the hydrogen peroxide concentration. Figure 6B shows an emission peak at 504 nm that increases with *c*H_2_O_2_. However, after reaching a maximum, the CL intensity apparently decreases at higher hydrogen peroxide concentrations (Figure 6C). This is attributed here to the fact that a single filter paper sensor was employed repeatedly, and the addition of H_2_O_2_ does not account for effects of reaction time and effects of TCPO reagent depletion/exhaustion at the interface.

Next, the time dependence of CL emission data in experiments was considered at different concentrations of H_2_O_2_. Figure 7A–C shows emission spectra decreasing with time. Interestingly, plots of ln­(intensity) versus ln­(time) in Figure 7D suggest a Cottrellian slope of approximately −0.5 consistent with diffusion control.^44^ This suggests that the concentration gradient of H_2_O_2_ into the microporous film could be limited by diffusion (and reaction, *vide infra*). With time, TCPO is consumed, the diffusion path lengthens, and therefore the luminescence weakens according to Cottrell’s inverse square root behavior.

**Figure 7:**
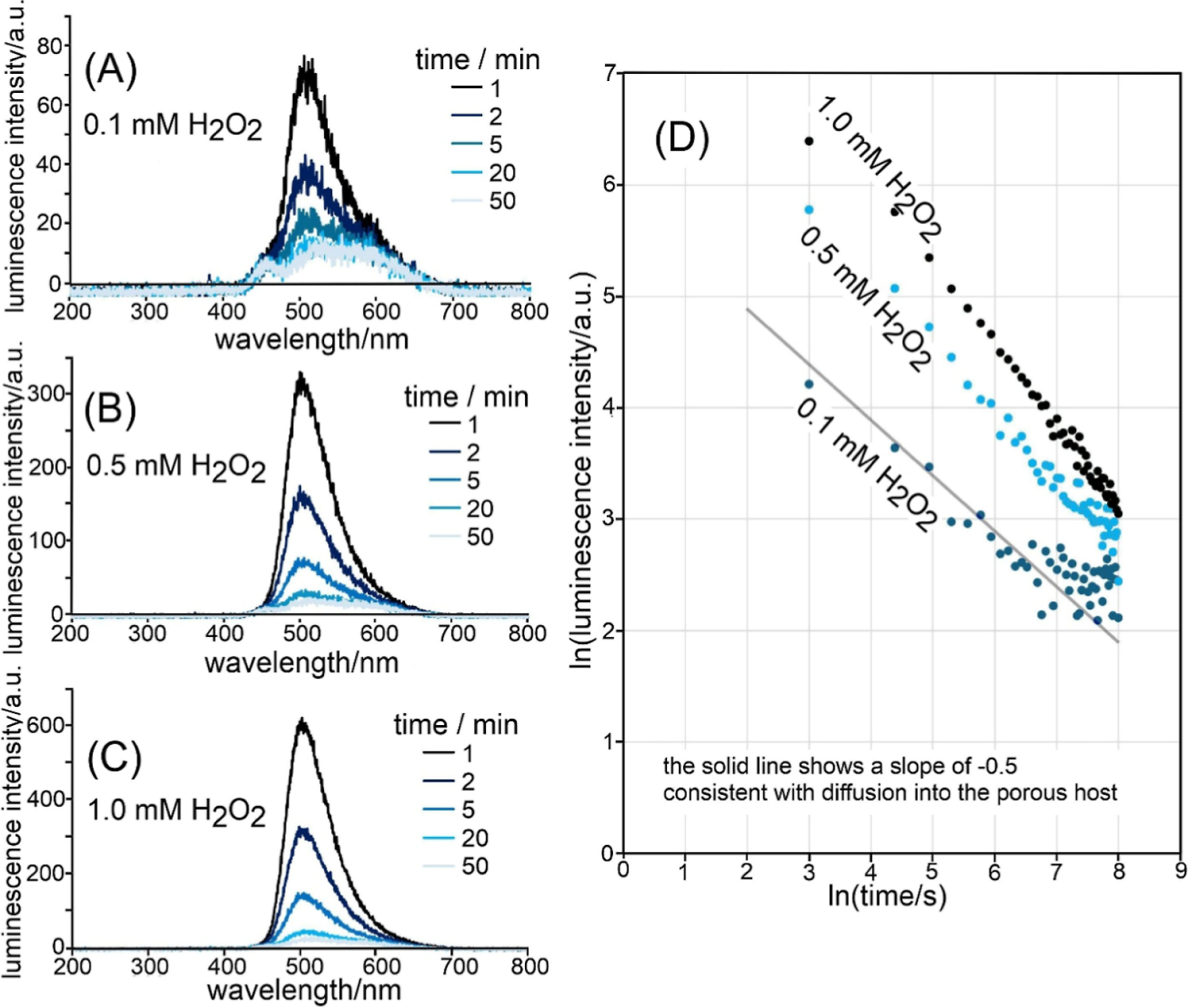
Chemiluminescence spectra for PIM-1/TCPO (at about 20 s; each 30 μg) on filter paper in 0.1 M imidazolium buffer pH 7 with (A) 0.1 mM, (B) 0.5 mM, (C) 1.0 mM H_2_O_2_. (D) Plot of ln­(CL intensity) versus ln­(time) with a line indicating slope −0.5 associated with diffusion into the microporous host (*vide infra*).

### 3.3 Observation and Optimization of Chemiluminescence (CL) Spectra for Glucose Detection with PIM-1/TCPO Deposits

Given the sensitivity of the chemiluminescence signal toward hydrogen peroxide, a calibration was performed by varying the hydrogen peroxide concentration and using a fresh filter paper for each measurement (Figure 8; for triplicate measurements typically ± 10% standard deviation). Errors here are mainly due to variation in the PIM-1/TCPO deposit and positioning of the filter paper (see Figure 3).

**Figure 8:**
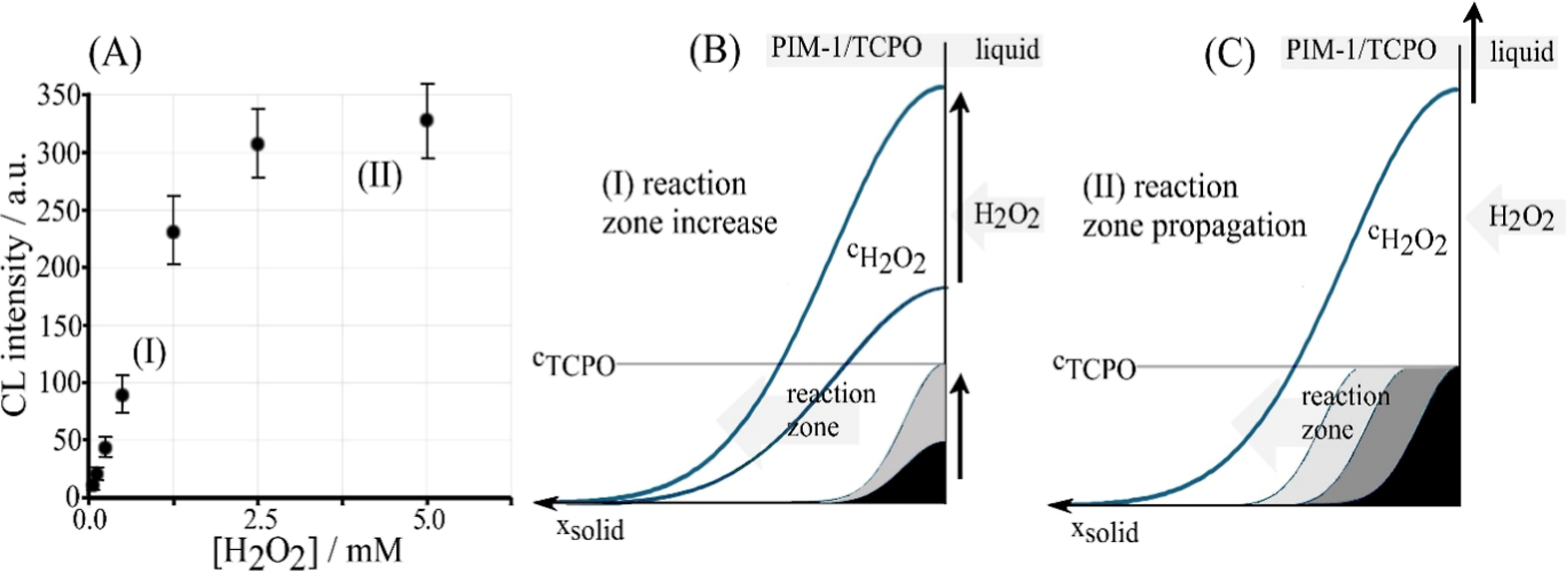
(A) Data for chemiluminescence intensity versus H_2_O_2_ concentration (after 20 s reaction; PMT voltage 600 V) with kinetic zones (I; reaction zone increases) and (II; reaction zone remains constant but propagates) indicated. For the range [H_2_O_2_] = 0.0–2.5 mM, a linear trendline is obtained with CL intensity = 91.739*x* + 0.6212 (*R* ^2^ = 0.9983 and LOD 10 μM) using 3 standard deviations of the blank. (B) Illustration for (I) the initial increase of chemiluminescence with [H_2_O_2_]. (C) Illustration for (II) the limit in chemiluminescence at high [H_2_O_2_].

Data in Figure 8 suggest that an approximately linear increase in intensity occurs at lower hydrogen peroxide concentrations, with a plateau at [H_2_O_2_] = 5 mM or higher. The observed behavior can be qualitatively assigned to H_2_O_2_ diffusion into the microporous material and formation of an emissive reaction zone. With an increase in [H_2_O_2_], the reaction zone is expanded and more TCPO is converted (see Figure 8B). However, for high [H_2_O_2_], the TCPO reagent is consumed/exhausted, and the reaction front must propagate further into the microporous material without a further increase in emission (see Figure 8C). The observed trend in the data is linked here to diffusion, although it could be compared with pseudo-Michaelis–Menten kinetics. Attempts (unwarranted) to fit data to Michaelis–Menten equations only give poor results, and therefore, better models are required (*vide infra*).

The production of H_2_O_2_ due to glucose oxidase reacting with glucose and oxygen was investigated (30 min incubation at room temperature; 20 s delay from immersion of the filter paper to recording luminescence data). Glucose oxidase (10 U mL^–1^) and glucose were used in a 2 mL volume, giving glucose concentrations of 1 to 10 mM in 0.1 M imidazolium buffer, pH 7. Glucose oxidase is known to catalyze the oxidation of glucose to gluconolactone while reducing ambient oxygen to hydrogen peroxide^45^ (eq 3).

3 $$D‐glucose+O_{2}\to glucono‐lactone+H_{2}O_{2}$$

Figure 9A shows detection of H_2_O_2_ correlated to the glucose concentration (after 30 min of reaction time). The limiting luminescence intensity of typically 70 au in Figure 9A corresponds to approximately 1 mM H_2_O_2_ in the calibration in Figure 8A. This limit (compared to the calibration data in Figure 8A) is likely to be imposed here due to limited flux and depletion of oxygen into the solution (rather than by any other kinetic limitations).

**Figure 9:**
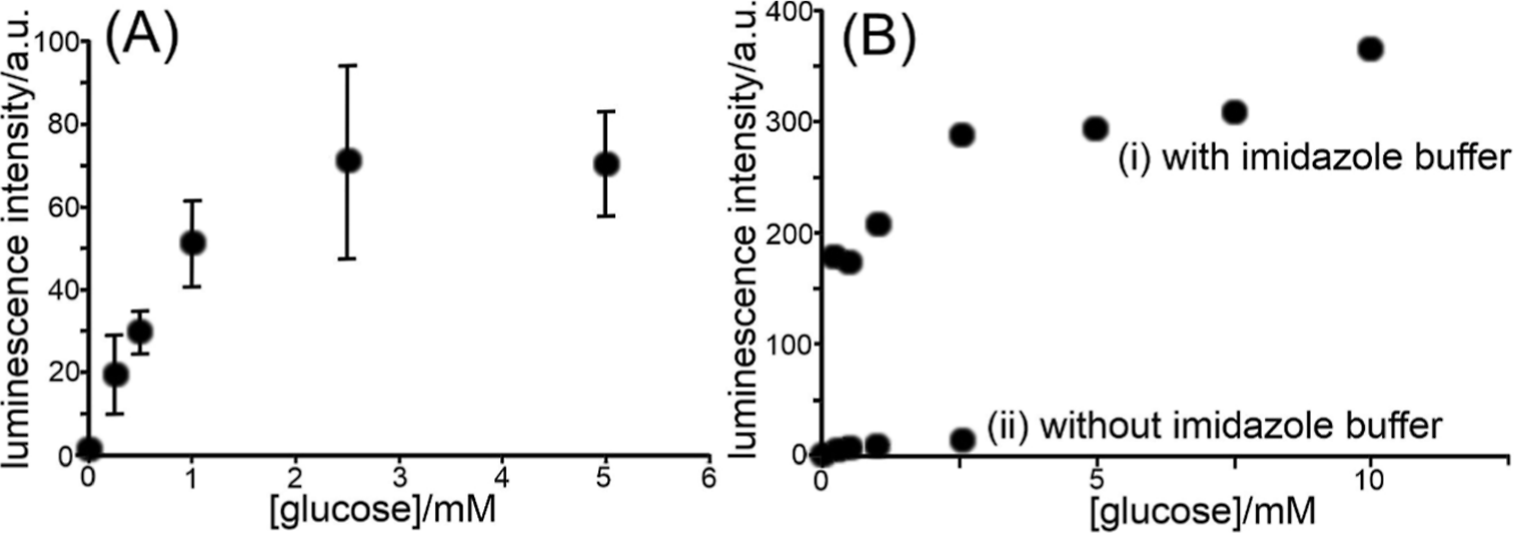
(A) Glucose detection based on 30 min incubation with glucose oxidase followed by chemiluminescence detection (PMT voltage 600 V; error bars based on triplicate measurements with fresh filter paper). (B) As before, but data for a single filter paper for each sequence of measurements comparing conditions (i) with 0.1 M imidazole buffer pH 7 and (ii) with 0.1 M phosphate buffer pH 7 (PMT 800 V).

In Figure 9B a comparison is shown for (i) chemiluminescence in imidazole buffer and (ii) chemiluminescence in phosphate buffer in the absence of imidazole (each obtained with a single filter paper). Perhaps surprisingly, imidazole is not essential but certainly helpful as the “trigger reagent” to improve the emission intensity. The emission is clearly much improved in the presence of imidazole, consistent with literature reports. In order to provide additional insight into the proposed mechanism, computer simulation experiments investigating diffusion and reaction in the microporous host environment are performed.

### 3.4 Simulation of Chemiluminescence (CL) Intensity for H_2_O_2_ Detection with PIM-1/TCPO Deposits

Digital simulation was performed in order to explore diffusion and reaction in microporous films during light emission (using a Fortran program in the MATLAB R2017b environment; see Supporting Information). A diffusion model was implemented as shown in Figure 10A (discrete boxes *x*_0_, *x*_1_, *x*_2_, ... *x*_*n*_) with finite difference expressions defined in eqs 4 and 5.

4 $$\frac{\partial c_{H_{2}O_{2}}}{\partial t}=D_{H_{2}O_{2}}\frac{\partial^{2}}{{\partial x}^{2}}c_{H_{2}O_{2}}-k_{c}c_{TCPO}c_{H_{2}O_{2}}$$

5 $$\frac{\partial c_{TCPO}}{\partial t}=D_{TCPO}\frac{\partial^{2}}{{\partial x}^{2}}c_{TCPO}-k_{c}c_{TCPO}c_{H_{2}O_{2}}$$

In these two equations, Fickian transport (one-dimensional^46^) and reactivity of H_2_O_2_ and TCPO are defined with the second-order reaction term *k*_c_ *c*_TCPO_ *c*H_2_O_2_ correlating to the light emission (and defining the loss of TCPO). Diffusivity parameters *D*_H_2_O_2__ = 10^–13^ m^2^ s^–1^ (*vide supra*) and *D*_TCPO_ = 10^–16^ m^2^ s^–1^ are chosen to reflect realistic diffusion rates for H_2_O_2_ in micropores (based on recent measurements for the O_2_ diffusion in PIM-1 ^35^) and to essentially halt diffusion for the much bigger TCPO molecules in the microporous environment. The model has a total length of 10 μm (to avoid effects from a finite diffusion space), and the size of individual boxes is d*x* = 0.01 μm, resulting in the number of boxes *n* = 1000 (see Supporting Information). The concentration for TCPO is fixed at both ends with *c*_TCPO_(*x*_0_) = *c*_TCPO_(*x*_*n*_) = 1000 mol m^–3^ (based on PIM-1/TCPO 1:1 weight ratio and the assumption of a density^47^ of 1 g cm^–3^). The concentration of hydrogen peroxide is fixed at the polymer|solution interface with typically *c*H_2_O_2_ (*x*_0_) = 1 mol m^–3^ and with an assumed no flux boundary for *c*H_2_O_2_ (x_*n*_). Diffusion in the aqueous solution phase and partitioning equilibria at the interface are ignored.

**Figure 10:**
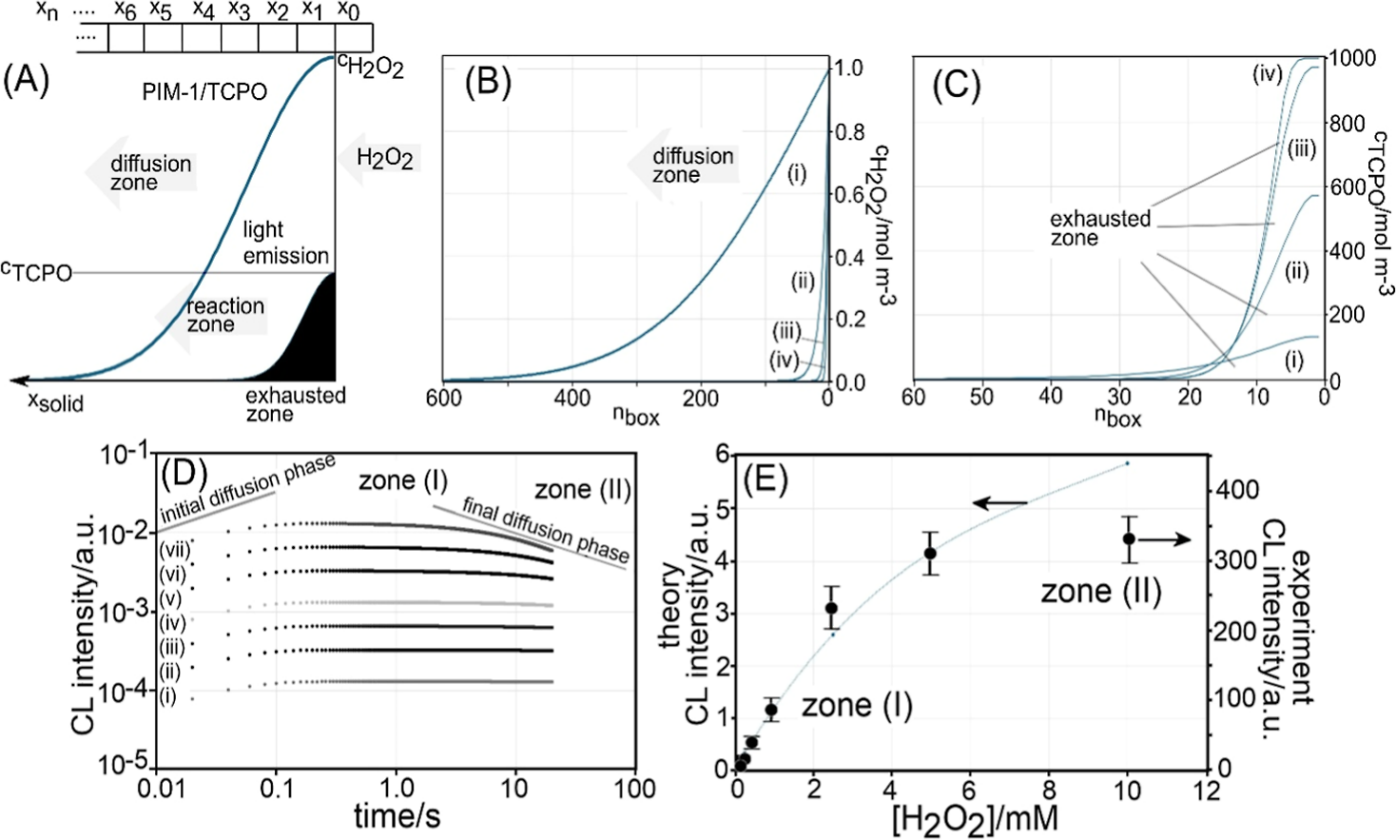
(A) Schematic for the one-dimensional computational model to treat diffusion and reaction in the microporous PIM-1/TCPO material. Boxes *x*_*n*_ indicate a 10 nm space with *n*_max_ = 1000, giving a 10 μm length. The diffusion reaction of H_2_O_2_ (eq 1) and TCPO (eq 2) are treated simultaneously. (B) Plot of *c*H_2_O_2_ versus depth for 20 s time and for *k*_c_ = (i) 10^–7^, (ii) 10^–6^, (iii) 10^–5^, and (iv) 10^–4^ mol^–1^m^3^ s^–1^. The faster the reaction, the less deep the diffusion process penetrates. (C) Plot of *c*_TCPO_ versus depth or *k*_c_ = (i) 10^–7^, (ii) 10^–6^, (iii) 10^–5^, and (iv) 10^–4^ mol^–1^m^3^ s^–1^. The faster the reaction, the more TCPO is consumed at the interface. (D) Plot of chemiluminescence intensity (CL) versus time for a simulation with initially *c*_TCPO_ = 1000 mol m^–3^, *c*_H_2_O_2__ = (i) 0.1, (ii) 0.25, (iii) 0.5, (iv) 1.0, (v) 2.5, (vi) 5.0, and (vii) 10 mol m^–3^, and *k*_c_ = 2 × 10^–7^ mol^–1^ m^3^ s^–1^. (E) Plot of chemiluminescence intensity (theory versus experiment) as a function of *c*H_2_O_2_ with (I) linear region of TCPO consumption and (II) interfacial exhaustion of TCPO linked to deeper diffusion.

Figure 10A shows the schematic for the model with hypothetical concentration gradients for H_2_O_2_ and for TCPO. Figure 10B shows the calculated concentration gradient for *c*_H_2_O_2__ at *t* = 20 s. Given the diffusion coefficient of *D*H_2_O_2_ = 10^–13^ m^2^ s^–1^, a diffusion layer of $\delta_{diffusion}=\sqrt{\pi D_{H_{2}O_{2}}t}$ = 2.5 μm can be predicted, and this is observed (see trace (i)) for very low second-order rate constants *k*_c_. When increasing the rate constant *k*_c_ to 10^–4^ mol^–1^ m^3^ s^–1^ (trace (iv)), the reaction layer dominates with $\delta_{reaction}=\sqrt{\frac{D_{H_{2}O_{2}}}{k_{c}}}$ = 3.2 nm. However, the TCPO reagent is consumed in this process, and therefore, the reaction layer will start to penetrate deeper into the microporous film. Figure 10C shows a plot of concentration *c*_TCPO_ for *t* = 20 s and *k*_c_ = (i) 10^–7^, (ii) 10^–6^, (iii) 10^–5^, and (iv) 10^–4^ mol^–1^ m^3^ s^–1^. The higher the second order rate constant, the deeper the reaction progresses into the film leading to an exhausted zone without TCPO.

The rate given by the second-order chemical reaction term in eqs 4 and 5 can be assumed to be proportional to the chemiluminescent light emission (assuming unit quantum yield and no further absorption of emitted light in thin films). It is, therefore, possible to calculate the chemiluminescence intensity as a function of time. Figure 10D shows plots of light emission versus time for seven concentrations of hydrogen peroxide. An initial rise in CL intensity can be attributed to diffusional transport into the microporous host (proportional to $\sqrt{t}$ ). The constant CL intensity is then proportional to *c*_H_2_O_2__ in the solution phase (see zone (I)). At longer times, TCPO consumption causes exhaustion, and the process again becomes diffusion controlled (see zone (II)) to move deeper into the microporous host (proportional to 1/ $\sqrt{t}$ ). The transition points of zone (I) and zone (II) are sensitive to the choice of rate constant *k*_c_. Figure 10E shows the original experimental data (Figure 8A) with theory data (line) superimposed. A reasonable/approximate agreement is obtained (see the solid line) for a second-order rate constant *k*_c_ = 2 × 10^–7^ mol^–1^ m^3^ s^–1^.

Although the PIM-1/TCPO film deposit on the filter paper is geometrically not well-defined, the features of the experimental and the theory data show good agreement, and the mechanistic interpretation can be assumed to be correct, at least in first approximation. In the future, the diffusion—reaction model could be improved to allow further microporous host systems or excited state-forming reagents to be compared.

According to the model, the polymer solid|electrolyte solution interface is the location of the light emission process, and therefore any methodology that increases the magnitude of this interface (*i.e*., using porous substrates or using nanoparticulate or electrospun PIM-1/TCPO) will potentially dramatically increase the light emission. The solid-state emission of (electro-)­chemiluminescence signals from microporous hosts will be useful in analytical challenges, where traces of hydrogen peroxide need detection without the addition of reagents, *e.g*., in tissue^48^ or in monitoring agricultural products such as the quality of milk.^49^

## 4 Conclusion and Outlook

Solid-state chemiluminescence and electrochemiluminescence were observed from a microporous polymer film (PIM-1) with embedded TCPO deposited on (i) graphene foam or (ii) filter paper. The process is triggered by TCPO reacting first with imidazole and second with hydrogen peroxide. The intensity of the light emission process from the PIM-1 host is directly proportional to the hydrogen peroxide concentration, but it saturates and even depletes at 5 mM or higher hydrogen peroxide concentrations due to interfacial consumption of the TCPO reagent. The time dependence of the light emission has been proposed to be associated with Cottrellian dynamics with an early phase of TCPO consumption close to the surface and a later phase of reaction deeper within the microporous host.

Key conclusions are (i) given that one-dimensional diffusion into the microporous host is limiting light emission, an increase in the surface area (e.g., using highly porous substrates or nanoparticles) should substantially increase light emission and therefore improve sensitivity; (ii) porosity changes (or hierarchical porosity) in the PIM-1 host will allow faster diffusion, and this also will enhance light emission; (iii) the need for imidazole as a “trigger” reagent could be circumvented in the future by coimmobilizing imidazole derivatives into the PIM-1 host film; (iv) a computational model can be employed to rationalize the diffusion-reaction processes at the interface of the microporous PIM-1/TCPO film and the aqueous phase. More work will be required to explore the effect of TCPO on PIM-1 microporosity and therefore on diffusion processes. Furthermore, better experimental methods such as *in situ* luminescence microscopy will be necessary to validate the proposed mechanistic model.

On graphene foam electrodes coated with PIM-1/TCPO, the reduction of oxygen to hydrogen peroxide is sufficient to trigger light emission (electrochemiluminescence) due to *in situ* formation of hydrogen peroxide at the electrode. For PIM-1/TCPO on filter paper substrates, the presence of hydrogen peroxide (e.g., from biosensor reactions) triggers chemiluminescence. In the future, other types of fluorescent PIM materials could be investigated and compared to further improve light emission and analytical performance, as well as eliminate the need for imidazole. Other types of excited state generating coreactants could be investigated. For biosensor applications, enzymes could be coimmobilized to give all-solid-state CL biosensor films.

## Supplementary Material



## Notes

Additional information provided in the Supporting Information are ATR–FTIR spectra, SEM images of graphene foam surfaces and of PIM-1/TCPO-coated graphene foam surfaces, SEM/EDS data for the bare graphene foam substrate, SEM for a cross-section of PIM-1/TCPO-coated graphene foam, SEM images of filter paper surfaces, SEM/EDS for the bare and coated filter paper, SEM cross-sectional image for a coated filter paper, cyclic voltammograms and voltlumograms for PIM-1/TCPO-coated graphene foam electrodes, and the Matlab simulation code (PDF)
