# Supplementary material for: Chemiluminescence Detection of Hydrogen Peroxide with a Polymer of an Intrinsic Microporosity Solid State Emitter
Source: ACS Appl Polym Mater. 2026 May 4;8(10):7657–67. doi: 10.1021/acsapm.6c00847 (PMC13200164; doi:10.1021/acsapm.6c00847)
Supplement: Supplementary file 1 [file ap6c00847_si_001.pdf]

SUPPORTING INFORMATION

Chemiluminescence Detection of Hydrogen Peroxide with a Polymer of Intrinsic Microporosity (PIM-1) Solid State Emitter

Supharada Phokhabut [1,2], Tinakorn Kanyanee [2,3]*, Michael Zachariadis [4], Silvia Martinez Micol [4], Philip J. Fletcher [4], Mariolino Carta [5,6], Dominic Taylor [7], Neil B. McKeown [7], Marco Caffio [8], Oliver Matys [1], Frank Marken [1]* (email f.marken@bath.ac.uk)

[1] *Department of Chemistry, University of Bath, Claverton Down, BA2 7AY, UK*

[2] *Department of Chemistry, Faculty of Science, Chiang Mai University, Chiang Mai 50200, Thailand*

[3] *Materials Science Research Center, and Center of Excellence for Innovation in Chemistry, Faculty of Science, Chiang Mai University, Chiang Mai, 50200, Thailand*

[4] *Imaging Facility, University of Bath, Bath BA2 7AY, UK*

[5] *Faculty of Science and Engineering, Department of Chemistry, Swansea University, College of Science, Grove Building, Singleton Park, Swansea SA2 8PP, UK*

[6] *Instituto de Síntesis Química y Catálisis Homogénea, CSIC-Universidad de Zaragoza, C/Pedro Cerbuna 12, Facultad de Ciencias, Zaragoza 50009, Spain*

[7] *EaStCHEM, School of Chemistry, University of Edinburgh, Joseph Black Building, David Brewster Road, Edinburgh, Scotland EH9 3JF, UK*

[8] *Integrated Graphene Ltd., Euro House, Wellgreen Place, Stirling FK8 2DJ, UK*

Content

Figure SI1. ATR-FTIR spectra for (A) filter paper; (B) filter paper with PIM-1 deposit; (C) filter paper with TCPO deposit; (D) filter paper with PIM-1/TCPO; (E) pure PIM-1; (F) pure TCPO; (G) graphene foam; (H) graphene foam with PIM-1/TCPO. **…………………….. S3**

Figure SI2. SEM images of graphene foam surfaces (A) bare and (B) coated with 60 μg of a mixture of PIM-1/TCPO (1:1)**…………………………………………………… S4**

Figure SI3. (A) Scanning electron microscopy (SEM) image for a PIM-1/TCPO coated graphene foam surface. (B-E) Energy dispersive x-ray spectroscopy (EDS) for a PIM-1/TCPO coated graphene foam surface. (F) Table of EDS data. **………………………………….. S4**

Figure SI4. (A) SEM/EDS for the bare graphene foam substrate. (B,C) Energy dispersive x-ray spectroscopy (EDS) for a bare graphene foam surface. (D) Table of EDS data. **……. S5**

Figure SI5. (A) Scanning electron microscopy (SEM) image for a cross-section of PIM-1/TCPO coated graphene foam. (B-E) Energy dispersive x-ray spectroscopy (EDS) for a graphene foam surface coated with 60 μg PIM-1/TCPO (1:1). (F) Table of EDS data. **… S5**

Figure SI6. SEM images of filter paper surfaces (A) bare and (B) coated with 60 μg PIM-1/TCPO (1:1). **……………………………………………………………………….. S6**

Figure SI7. SEM/EDS for the bare filter paper. **………………………………………. S7**

Figure SI8. SEM/EDS for filter paper coated with 60 μg PIM-1/TCPO (1:1). **…………. S7**

Figure SI9. (A) Scanning electron microscopy (SEM) cross-sectional image for a filter paper with 60 μg PIM-1/TCPO (1:1). (B-E) Energy dispersive x-ray spectroscopy (EDS) for a filter paper surface coated with 60 μg PIM-1/TCPO (1:1). (F) Table of EDS data. **…………… S8**

Figure SI10. Cyclic voltammograms (A) and voltlumograms (B) for PIM-1/TCPO coated graphene foam electrodes (PMT voltage 800 V; scan rate 20 mV s^{-1}) in 1 M imidazolium buffer (i) pH 7, (ii) pH 8, and (iii) pH 9. **……………………………………………………… S9**

Matlab simulation code ………………………………………………………………… S10

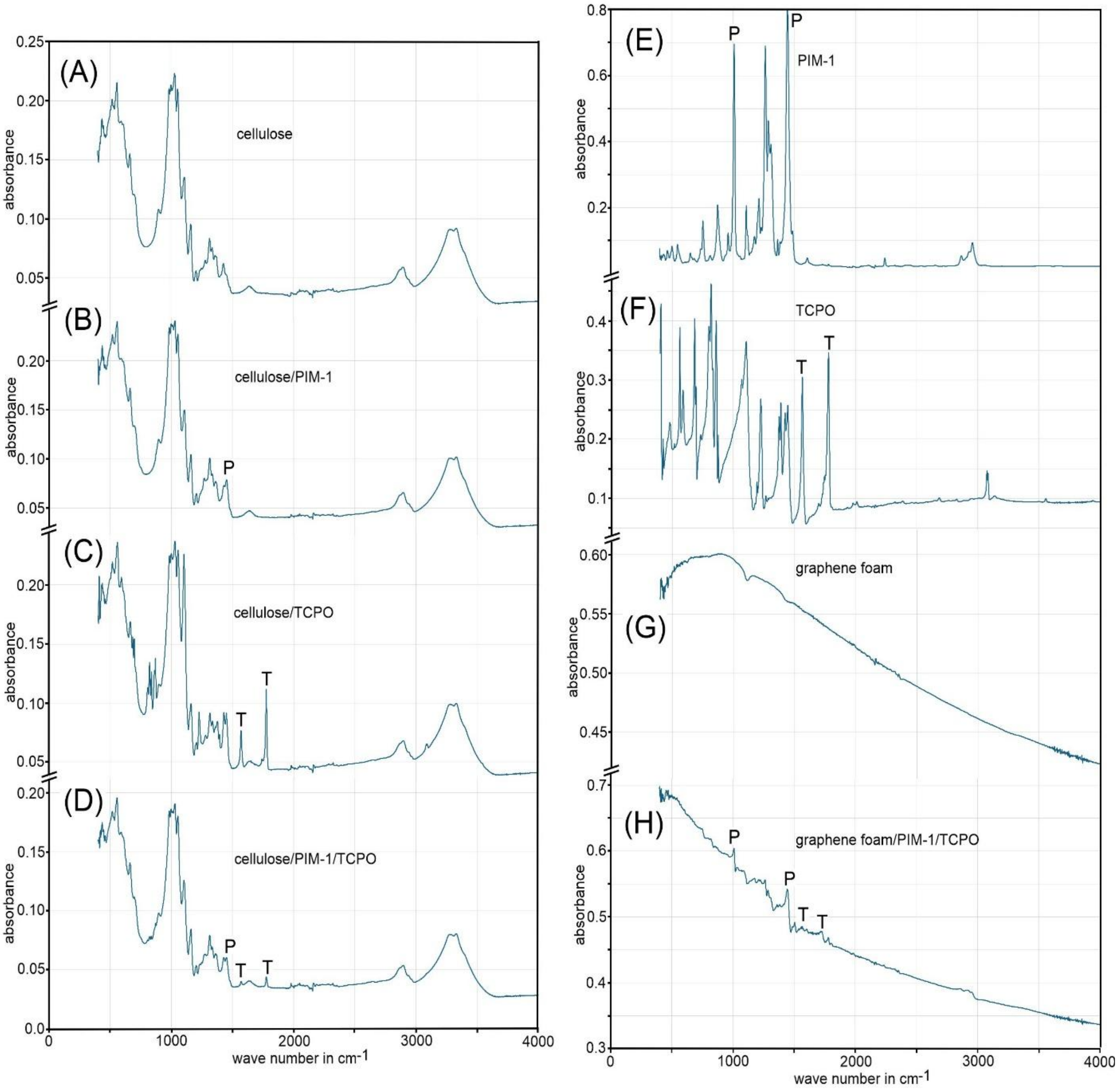

Figure SI1. ATR-FTIR spectra for (A) filter paper; (B) filter paper with PIM-1 deposit; (C) filter paper with TCPO deposit; (D) filter paper with PIM-1/TCPO; (E) pure PIM-1; (F) pure TCPO; (G) graphene foam; (H) graphene foam with PIM-1/TCPO. Indicator peaks for TCPO shown as “T” and indicator peaks for PIM-1 shown as “P”.

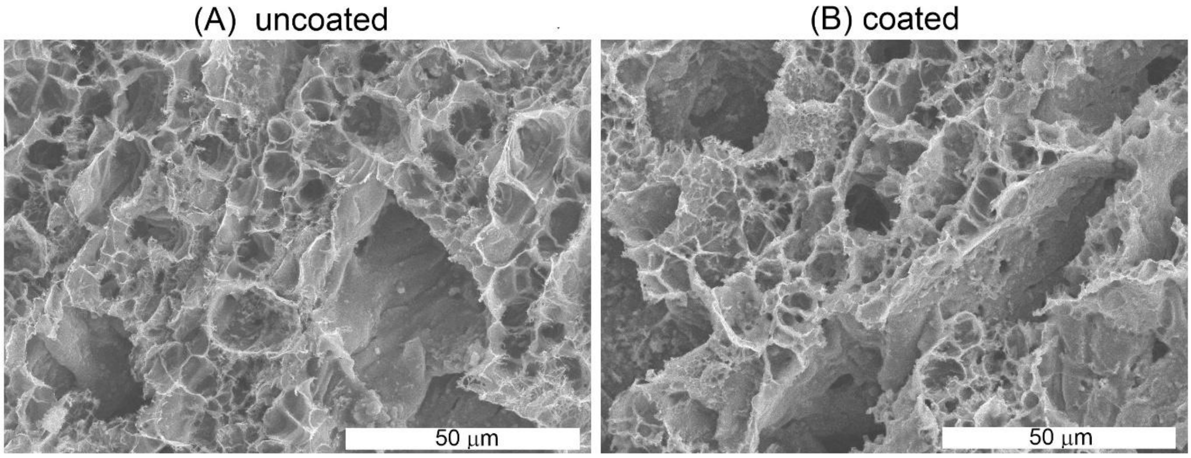

Figure SI2. SEM images of graphene foam surfaces (A) bare and (B) coated with 60 μg of a mixture of PIM-1/TCPO (1:1).

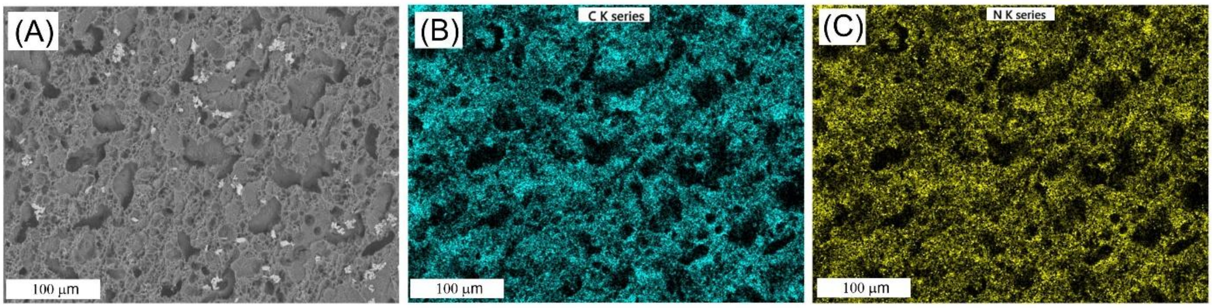

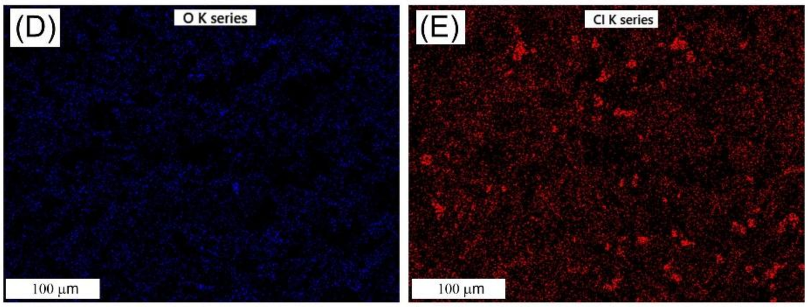

(F)

Element			Wt%	Wt% Sigma	Atomic %
C	EDS	K series	87.73	0.17	93.82
N	EDS	K series	0.11	0.17	0.10
O	EDS	K series	3.81	0.06	3.06
Cl	EDS	K series	8.34	0.06	3.02
Total			100.00		100.00

Figure SI3. (A) Scanning electron microscopy (SEM) image for a PIM-1/TCPO coated graphene foam surface. (B-E) Energy dispersive x-ray spectroscopy (EDS) for a PIM-1/TCPO coated graphene foam surface. (F) Table of EDS data.

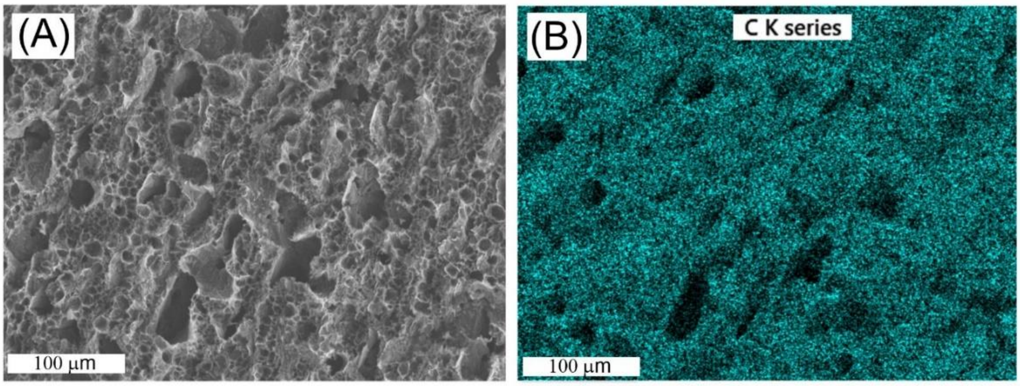

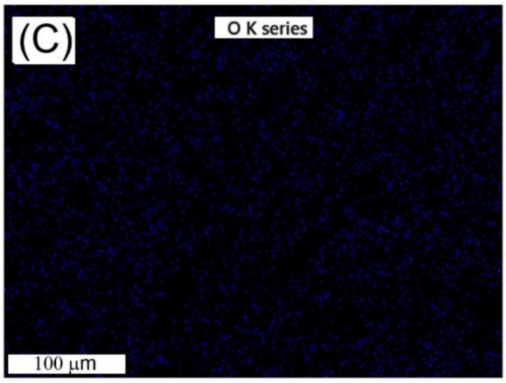

(D)

Element		Line	Wt%	Wt% Sigma	Atomic %
B	EDS	K series	0.00	0.31	0.00
C	EDS	K series	96.95	0.10	97.69
N	EDS	K series	0.00	0.29	0.00
O	EDS	K series	3.05	0.10	2.31
Total			100.00		100.00

Figure SI4. (A) SEM/EDS for the bare graphene foam substrate. (B,C) Energy dispersive x-ray spectroscopy (EDS) for a bare graphene foam surface. (D) Table of EDS data.

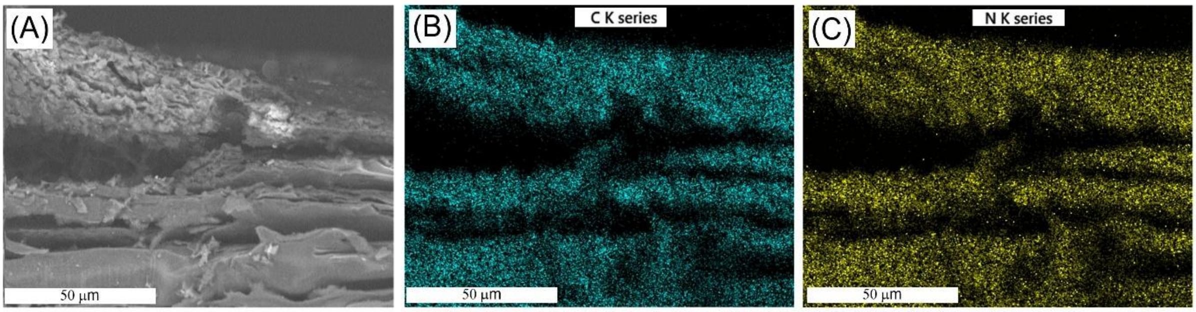

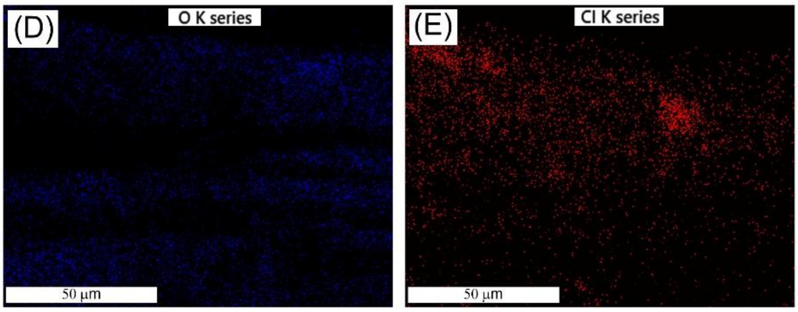

(F)

Element			Wt%	Wt% Sigma	Atomic %
C	EDS	K series	77.31	0.44	83.21
N	EDS	K series	3.54	0.48	3.27
O	EDS	K series	14.67	0.22	11.85
Al	EDS	K series	0.33	0.04	0.16
Cl	EDS	K series	4.16	0.10	1.52
Total			100.00		100.00

Figure SI5. (A) Scanning electron microscopy (SEM) image for a cross-section of PIM-1/TCPO coated graphene foam. (B-E) Energy dispersive x-ray spectroscopy (EDS) for a graphene foam surface coated with 60 µg PIM-1/TCPO (1:1). (F) Table of EDS data.

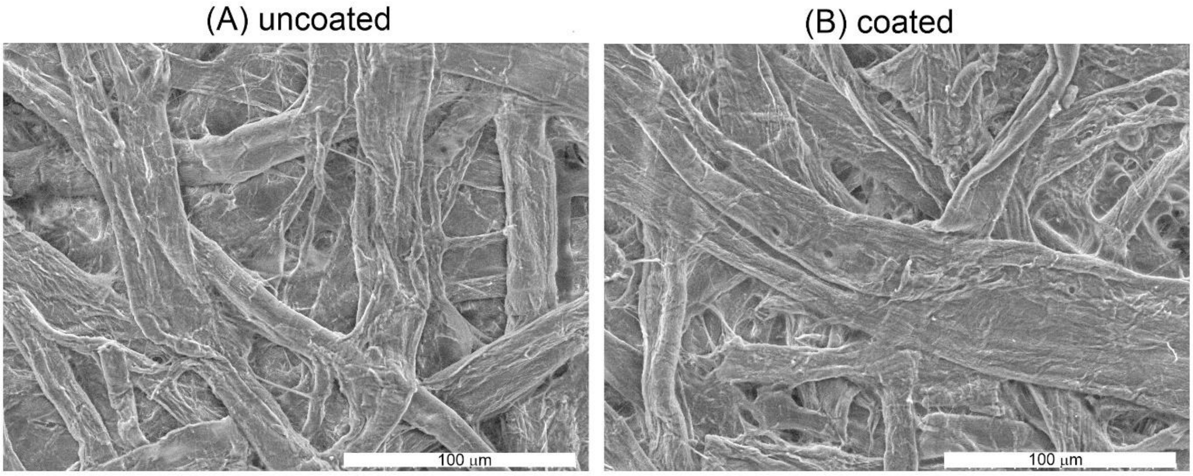

Figure SI6. SEM images of filter paper surfaces (A) bare and (B) coated with 60 μg PIM-1/TCPO (1:1).

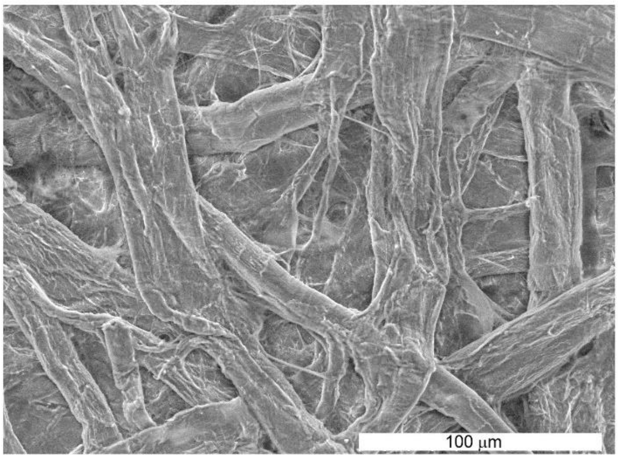

Element	Signal Type	Line	Wt%	Wt% Sigma	Atomic %
C	EDS	K series	48.80	0.11	55.95
N	EDS	K series	0.03	0.10	0.03
O	EDS	K series	51.09	0.11	43.98
Al	EDS	K series	0.07	0.02	0.04
Cl	EDS	K series	0.00	0.02	0.00
Total			100.00		100.00

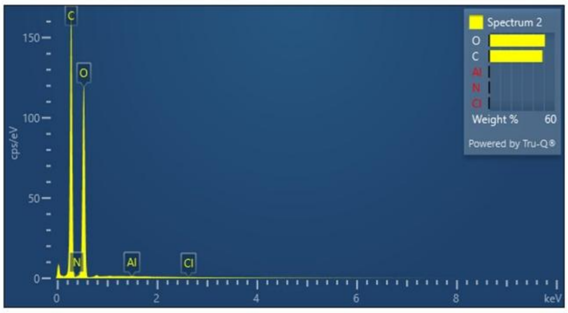

Figure SI7. SEM/EDS for the bare filter paper.

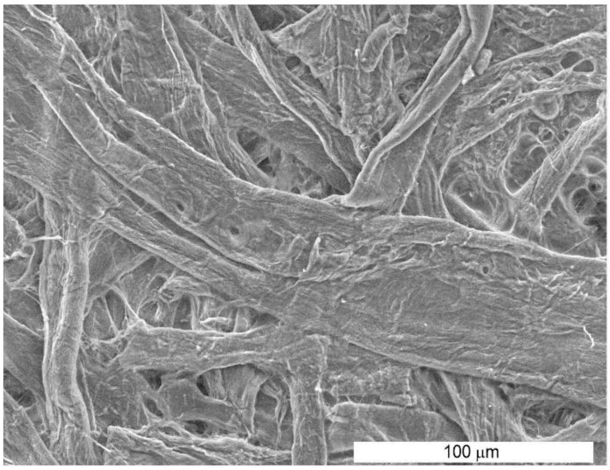

Element	Signal Type	Line	Wt%	Wt% Sigma	Atomic %
C	EDS	K series	50.01	0.11	57.25
N	EDS	K series	0.03	0.11	0.03
O	EDS	K series	49.48	0.11	42.53
Cl	EDS	K series	0.49	0.02	0.19
Total			100.00		100.00

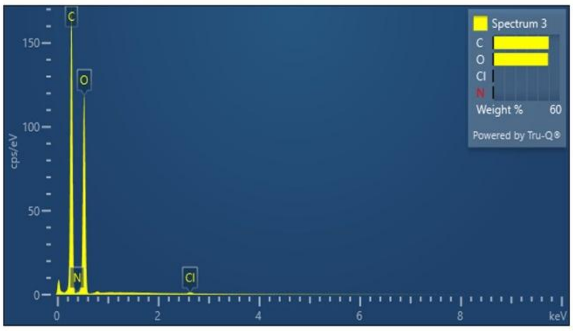

Figure SI8. SEM/EDS for filter paper coated with 60 μg PIM-1/TCPO (1:1).

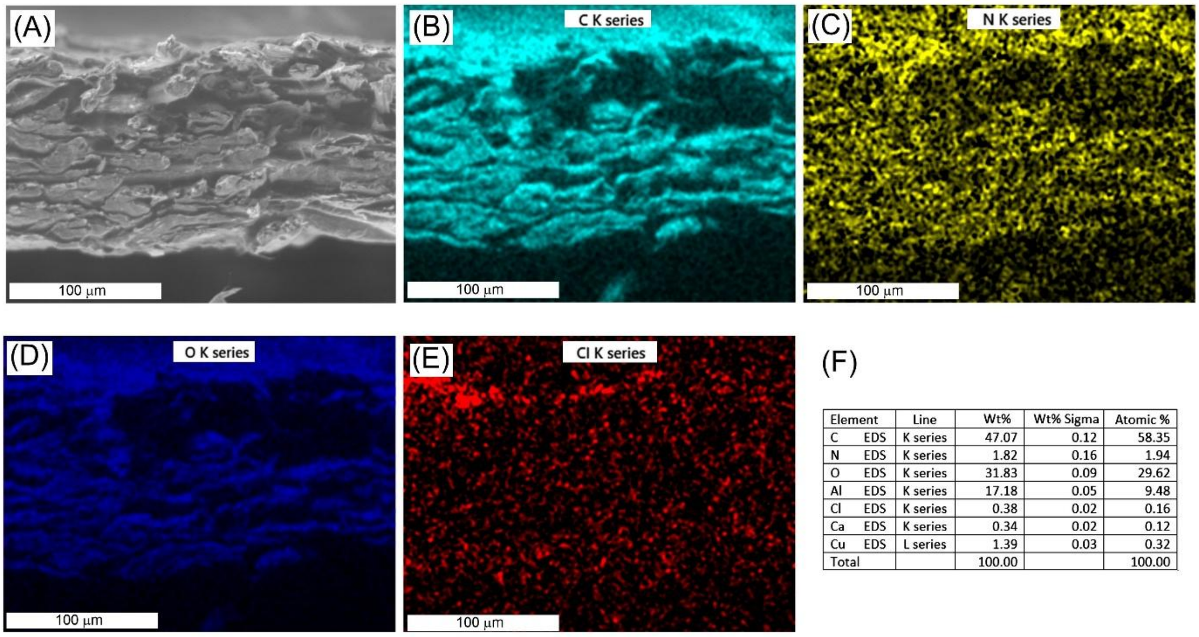

(F)

Element		Line	Wt%	Wt% Sigma	Atomic %
C	EDS	K series	47.07	0.12	58.35
N	EDS	K series	1.82	0.16	1.94
O	EDS	K series	31.83	0.09	29.62
Al	EDS	K series	17.18	0.05	9.48
Cl	EDS	K series	0.38	0.02	0.16
Ca	EDS	K series	0.34	0.02	0.12
Cu	EDS	L series	1.39	0.03	0.32
Total			100.00		100.00

Figure SI9. (A) Scanning electron microscopy (SEM) cross-sectional image for a filter paper with 60 μg PIM-1/TCPO (1:1). (B-E) Energy dispersive x-ray spectroscopy (EDS) for a filter paper surface coated with 60 μg PIM-1/TCPO (1:1). (F) Table of EDS data.

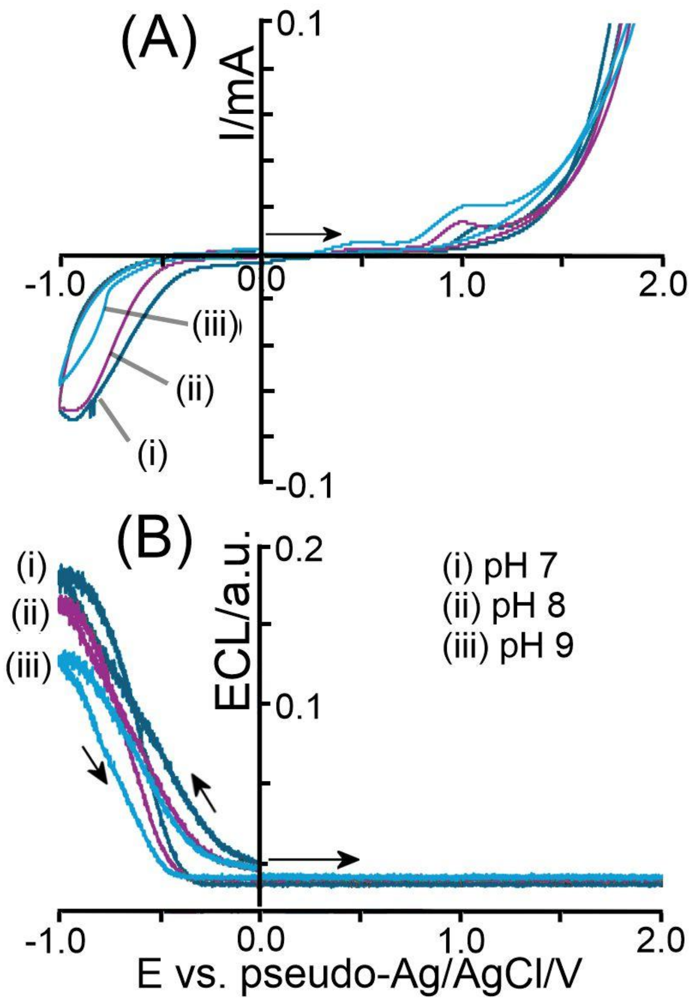

Figure SI10. Cyclic voltammograms (A) and voltlumograms (B) for PIM-1/TCPO coated graphene foam electrodes (PMT voltage 800 V; scan rate 20 mV s^{-1}) in 1 M imidazolium buffer (i) pH 7, (ii) pH 8, and (iii) pH 9.

Matlab simulation code

```
% Chemiluminescence within a PIM-1/TCPO layer 2026

clear;
L = 10e-6;          % total length
nbox = 1000;        % number of boxes
dt = 1e-5;          % time interval
time = 20;          % time in s for the simulation
ntime = time/dt;
npoints = 1000;     % limit data points with time
nskip = round(ntime/npoints);
dx = L/nbox;
DH2O2 = 1e-13;   % diffisuon coefficient for hydrogen peroxide
DTCPO = 1e-16;   % diffusion coefficient for TCPO
lambdaH2O2 = DH2O2*dt/dx/dx;
lambdaTCPO = DTCPO*dt/dx/dx;
cH2O2out = 10;   % fixed concentration of H2O2 at interface in mol m-3
kc = 2e-7;          % second order chemical rate constant in mol-1 m3 s-1
output(1:npoints,1:3) = 0;
counter = 0;

% initial conditions
for jx=1:nbox
   cHP(jx) = 0;
   cHPo(jx) = 0;
   cTCPO(jx) = 1000;
end

% time step one forward
  % time loop

  for jt=1:ntime
     cHPo = cHP;
     cTCPOo = cTCPO;
     cHP(1) = cH2O2out;
     emission = 0;
     for jx=2:nbox-1
        cHP(jx) = cHPo(jx) + lambdaH2O2*(cHPo(jx+1)-2*cHPo(jx)+ cHPo(jx-1))-
        kc*cHPo(jx)*cTCPOo(jx);
        cTCPO(jx) = cTCPOo(jx) + lambdaTCPO*(cTCPOo(jx+1)-2*cTCPOo(jx)+
        cTCPOo(jx-1))-kc*cHPo(jx)*cTCPOo(jx);
```

```
      emission = emission + kc*cHPo(jx)*cTCPOo(jx);
   end
   cHP(nbox) = 0;
   cTCPO(1) = cTCPO(2);

   if rem(jt,nskip) == 0
      counter = counter + 1;
      output(counter,1) = counter;
      output(counter,2) = nskip * counter * dt;
      output(counter,3) = emission;
   end
end
```
